# Mixed adenoma-neuroendocrine tumor of the stomach: analysis of nine cases with literature review

**DOI:** 10.1007/s00428-024-03851-3

**Published:** 2024-06-26

**Authors:** Hirofumi Rokutan, Miyako Fukasawa-Hokazono, Yukio Hokazono, Tetsuo Ushiku

**Affiliations:** 1 Department of Pathology, Graduate School of Medicine, The University of Tokyo, 7-3-1 Hongo, Bunkyo-Ku, Tokyo, 113-0033 Japan; 2 Department of Pathology, Yaizu City Hospital, Shizuoka, Japan

**Keywords:** Composite tumor, Stomach, Mixed adenoma-neuroendocrine tumor, Indolent

## Abstract

Mixed adenoma-neuroendocrine tumor (MANET) comprises adenoma and well-differentiated neuroendocrine tumor (NET) components. Given the limited information on this due to its rarity, we aimed to clarify the clinicopathologic features and optimal management of gastric MANETs in a case series and literature review. Nine patients with gastric MANETs, including eight male and one female patient (mean age, 72 years), were identified from the institutional pathology archive. Endoscopically, the tumors appeared as flat elevated lesions with sizes ranging from 0.8 to 4.4 cm. One patient had familial adenomatous polyposis, and no patient had autoimmune gastritis. All MANETs developed in the gastric body mucosa exhibiting chronic metaplastic atrophic gastritis. The glandular components were intestinal-type low-grade adenoma, and focal high-grade dysplasia was also recognized in three cases. The NET component was in middle/deep lamina propria in six cases and confined to deep lamina propria in the remaining three cases. Minimal cytologic atypia was found in the NET component, with no recognizable mitosis and a Ki-67 labeling index of < 2%. The NET component mostly showed diffuse positivity for serotonin and CDX2, suggesting that it consists of enterochromaffin cells. Diffuse p53 immunostaining was observed only in the high-grade adenomatous component of one case. No recurrence was observed during the follow-up period of 2–94 months. Correct distinction between the NET and poorly differentiated carcinoma components is crucial to prevent overtreatment of gastric MANETs. Considering its indolent nature, endoscopic resection is the primary recommendation for gastric MANETs as well as for pure adenomas.

**Supplementary Information:**

The online version contains supplementary material available at 10.1007/s00428-024-03851-3.

## Introduction

Mixed adenoma-neuroendocrine tumor (MANET) was initially proposed in 2012 to define colonic neoplasms containing both adenomatous and well-differentiated neuroendocrine tumor (NET) components [1]. According to the 2019 World Health Organization (WHO) classification of tumors of the digestive system [2], MANET is an entity not included in the category of mixed neuroendocrine-nonneuroendocrine neoplasm (MiNEN). Although the vast majority of MANETs have been reported in the colon, they also occur, albeit less frequently, in the stomach, duodenum, and ileum [3].

One study reported that the incidence of colonic MANETs was 3.8% (6 of 158) among surgically resected adenomas [4]. Colonic MANETs are likely to exhibit low malignant potential because of the indolent clinical course and the absence of cancer-related deaths in patients diagnosed with colonic MANETs [3]. Histologically, colonic MANETs are composed of tubular or tubulovillous adenoma components associated with WHO grade 1 or 2 NET. Some patients with colonic MANETs had familial adenomatous polyposis (FAP) syndrome [5], and intriguingly, a subset of MANETs exhibited β-catenin nuclear immunoreactivity or squamous morule formation, suggesting a pathogenic role for the Wnt/β-catenin signaling pathway in colonic MANETs [4, 5].

Conversely, the clinicopathologic features and the biologic behavior of gastric MANETs have not been well characterized due to their extreme rarity; only nine patients with gastric MANETs have been described in case reports or small case series in the literature [3, 6–10]. In the present study, we performed clinicopathologic analyses of nine patients with gastric MANETs to improve our understanding of the characteristics of this under-recognized entity.

## Materials and methods

### Clinical data

We reviewed all resected and biopsy specimens of gland-forming intramucosal neoplasms of the stomach in the University of Tokyo Hospital between January 1, 2005, and June 30, 2020 (*n* = 1559), and identified nine non-invasive gland-forming neoplasms mixed with NET components. Clinical and follow-up data and endoscopic findings were obtained from the medical records. Macroscopic tumor type was classified according to the Japanese classification of gastric carcinoma for early gastric cancer [11].

This study was approved by the Institutional Review Board of the University of Tokyo (approval no, G3521) and informed consent was obtained.

### Histologic assessment

The histopathologic features of hematoxylin/eosin-stained sections, which were available for all cases, were evaluated in detail by three pathologists (HR, MF-H, and TU). The features analyzed in the present study included sizes of the adenomatous and NET components, grade of the adenomatous (low-grade or high-grade) and NET (G1, G2, or G3) components according to the current WHO criteria [2], presence of the adenocarcinoma component, number of clusters/nests of the NET component in the highest field (× 400 magnification), distribution of the NET component within the lesion, and background mucosal condition.

### Immunohistochemical analysis

In all nine cases, formalin-fixed paraffin-embedded tissue blocks were available for immunohistochemistry. To determine tumor phenotype (i.e., the expression of gastric and intestinal phenotypic markers as well as neuroendocrine markers), NET cell type, and alterations in p53 and β-catenin expression, immunohistochemical staining was performed using antibodies listed in Table 1. The Ventana BenchMark ULTRA platform (Roche, Basel, Switzerland) was used according to the manufacturer’s protocols.

**Table 1:** List of antibodies used for immunohistochemistry

Antibody	Clone	M/P	Dilution	Source
Chromogranin A	DAK-A3	M	1:200	Dako
Synaptophysin	MRQ-40	M	Prediluted	Roche Tissue Diagnostics
Ki67	MIB1	M	1:200	Dako
p53	DO-7	M	1:50	Leica Biosystems
β-catenin	14/Beta-catenin	M	1:1000	BD Biosciences
MUC2	Ccp58	M	1:100	Santa Cruz biotechnology
MUC5AC	CLH2	M	1:200	Santa Cruz biotechnology
MUC6	CLH5	M	1:100	Santa Cruz biotechnology
CD10	56C6	M	1:200	Leica Biosystems
CDX2	CDX2-88	M	1:200	BioGenex
Serotonin	5HT-H209	M	1:1	Dako
Gastrin	7G9C3	M	1:1000	Proteintech
Somatostatin	-	P	1:1	Nichirei Biosciences
SSTR2A	UMB1	M	1:1000	Abcam
VMAT2	OTI9E11	M	1:500	Novus Biologicals

*M*, monoclonal; *P*, polyclonal

The slides stained for p53 were defined to exhibit mutant p53 pattern when strong nuclear staining was observed in more than 70% of all tumor cells or p53 staining was completely absent; all other staining patterns were defined as wild-type pattern, in which p53-positive and p53-negative cells were admixed with tumor cells [12]. Cases with nuclear β-catenin staining in > 10% of the tumor cells were categorized as β-catenin-positive.

Tumor cell phenotypes of the adenomatous component were defined by immunostaining for gastric (MUC5AC and MUC6) and intestinal (MUC2, CD10, and CDX-2) phenotypic markers, as previously described [13]. The tumor was defined as positive for each marker if more than 10% of the tumor cells were positive for the interrogated marker. Tumors positive for both gastric and intestinal markers were classified as mixed-type tumors. Two pathologists (HR and MF-H) evaluated all immunostained specimens.

## Results

### Clinical characteristics

The clinical characteristics of nine patients included in the study are summarized in Table 2. Briefly, the cohort included eight male and one female patient, with a mean age of 72.1 years (range, 58–80 years). Among the patients tested for serum *H. pylori* IgG antibody (*n* = 6), five (83%) were positive for the antibody. In one patient who was not tested for the serum antibody, *H. pylori* was detected in gastric specimen pathological examination. Taken together, six patients were confirmed to be infected by *H. pylori*. None of the patients had a history of long-term proton pump inhibitor (PPI) use or clinical features suggesting of autoimmune gastritis. Serum gastrin levels were not measured. One tumor (case 5) was from a patient with FAP, whereas the remaining tumors were not associated with a genetic predisposition such as FAP, multiple endocrine neoplasia (MEN), or Lynch syndrome.

**Table 2:** Clinical information of the patients included in the study

Case	Age (years)	Sex	Locus	Treatment	Endoscopic type	Size (mm)	*H. pylori* IgG	History of PPI
1	61	M	Body	ESD	Flat elevated	35	+	−
2	58	M	Body	ESD	Flat elevated	32	+	−
3	80	F	Fundus	ESD	Flat elevated	15	NA	−
4	64	M	Body	ESD	Flat elevated	44	−	11 weeks
5	77	M	Body	ESD	Flat elevated	15	+	37 weeks (quitted 6 years prior to ESD)
6	79	M	Body	ESD	Flat elevated	14	+	7 weeks (quitted 5 years prior to ESD)
7	80	M	Body	ESD	Flat elevated	13	NA (+ in gastric specimen)	−
8	75	M	Fundus	Subtotal esophagectomy	Flat elevated	27	+	−
9	75	M	Antrum	Biopsy	Flat elevated	8	NA	−

*ESD*, endoscopic submucosal dissection; *NA*, not assessed; *PPI*, proton pump inhibitor

The tumors were mostly (6/9) located in the body of the stomach. Endoscopically, all nine tumors appeared as flat elevated lesions. The tumor size ranged from 0.8 to 4.4 cm.

Endoscopic submucosal dissection was performed in seven patients, and one tumor was surgically resected. In one patient (case 9), only biopsy specimens were available, because the patient died due to another disease before gastric tumor resection.

In cases 4 and 7, the time interval between diagnosis and resection was long (60 and 52 months, respectively). During this period, there was a minimal change in tumor size between the first examination and resection in case 7, whereas the tumor increased from 3 to 4 cm in case 4.

### Pathologic features

The histologic features of all nine cases are summarized in Table 3. In all cases, tumors were intramucosal lesions with a superficially localized adenoma component and a more deeply localized NET component. The adenomatous component was composed of columnar epithelium with elongated nucleus and pseudostratification in all nine cases. The adenomatous component was uniformly low-grade in six cases but was adenoma exhibiting predominantly low-grade and focal high-grade dysplasia in the remaining three cases.

**Table 3:** Pathologic findings of the cases in the present study

Case	Glandular component		NET component											
	Mucin phenotype	Ki67	Size (mm)	Distribution	Grade	Number of cluster/HPF^#^	Ki67	SSTR2A	CDX2	VMAT2	Serotonin	Gastrin	Somatostatin	Cell type
1	Mixed	30%	17	Basal–middle	G1	18	1%	+	+	−	+	−	−	Serotonin-producing EC-cell
2	Mixed	5%	28	Basal–middle	G1	36	1%	+	+	−	+	−	−	Serotonin-producing EC-cell
3	NA*	NA*	4	Deep lamina propria	G1	14	NA*	NA*	NA*	NA*	NA*	NA*	NA*	NA*
4	Intestinal	30%	22	Deep lamina propria	G1	24	1%	+	+	−	+	−	−	Serotonin-producing EC-cell
5	Intestinal	5%	3	Deep lamina propria	G1	38	0.5%	+	+	−	+	−	−	Serotonin-producing EC-cell
6	Intestinal	30%	11	Basal–middle	G1	23	0.5%	+	+	−	−	−	−	EC-cell, unclassifiable
7	Intestinal	30%	12	Basal–middle	G1	23	1%	+	+	−	+	−	−	Serotonin-producing EC-cell
8	Intestinal	30%	14	Basal–middle	G1	46	1%	+	+	−	+	−	−	Serotonin-producing EC-cell
9	Intestinal	2%	NA	Basal–middle	G1	5	0.5%	NA**	NA**	NA**	NA**	NA**	NA**	NA**

*EC-cell*, enterochromaffin cell; *NA*, not available

^*^Inappropriate immunostaining results due to the sample condition in case 3

^**^It was unable to perform additional immunohistochemistry in case 9 (biopsy specimen)

^#^Number of clusters/nets of the NET component in the highest field (× 400 magnification) is presented

**Fig. 1:**
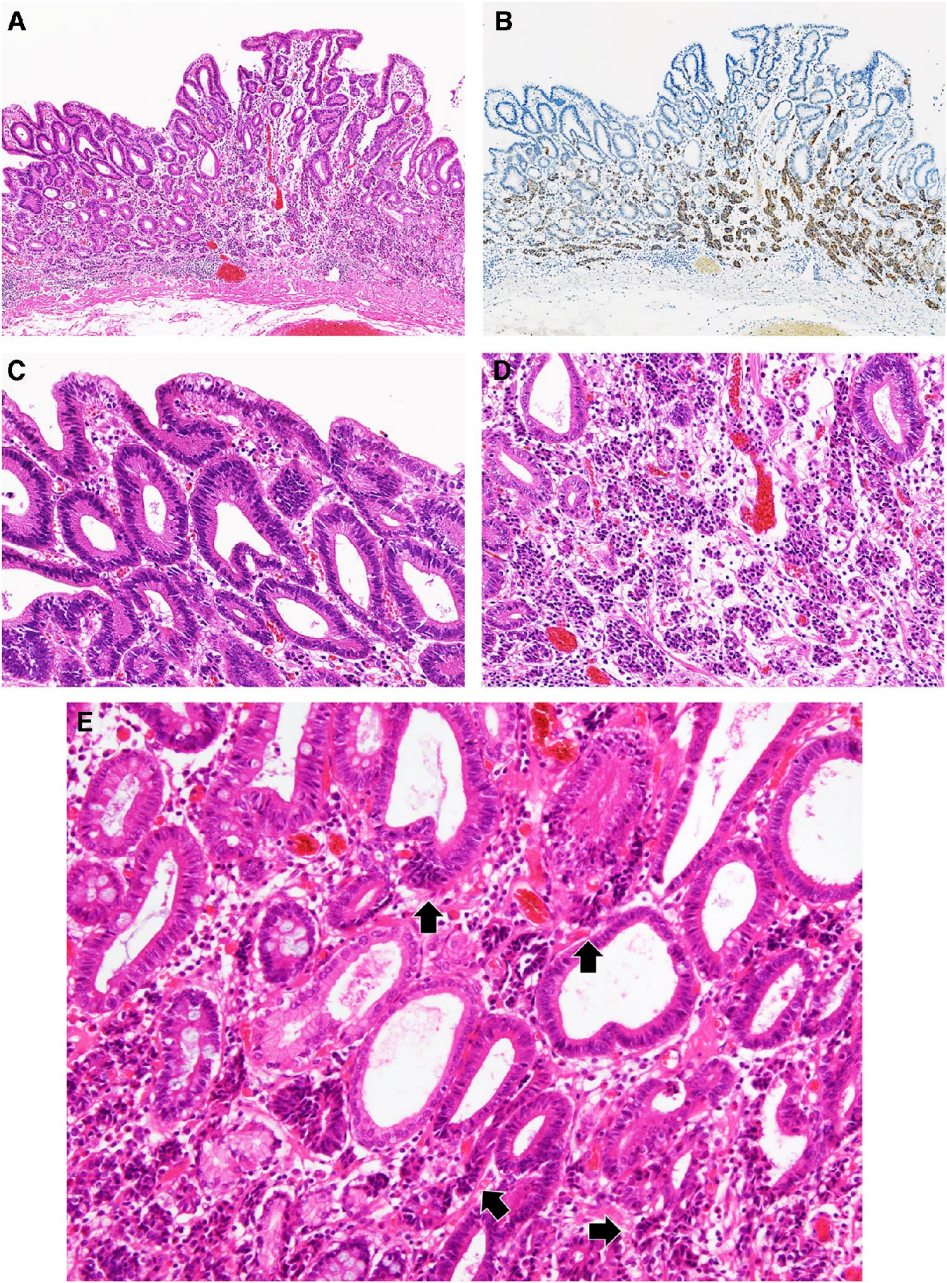
Representative images of gastric MANET, basal–middle pattern. **A**, **B** The neuroendocrine tumor (NET) component is seen in the middle/deep layer of lamina propria (basal–middle pattern) (**A**). Synaptophysin staining (**B**) highlights the NET component. **C** Higher magnification of the adenomatous component. **D** Higher magnification of the NET component forming small nests. **E** Note that clusters of neuroendocrine cells (arrows) are protruding from adenomatous glands. (**A**–**D** case 2; **E** case 1). MANET, mixed adenoma-neuroendocrine tumor

**Fig. 2:**
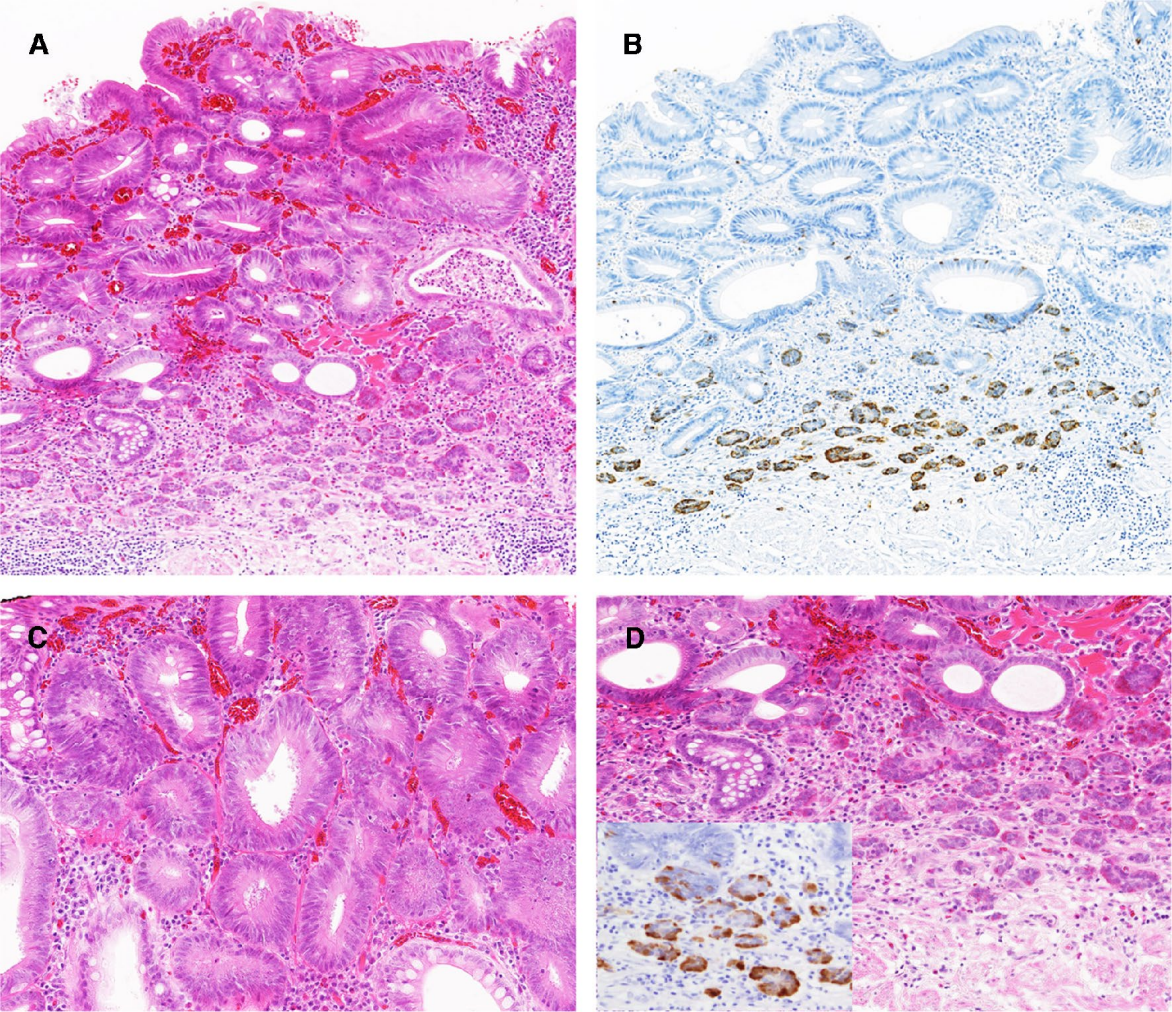
Representative images of gastric MANET, deep lamina propria pattern. **A** The NET component is confined to deep lamina propria (deep lamina propria pattern). **B** Chromogranin A staining highlights the NET component in deep lamina propria. **C** Higher magnification of the adenoma component (low-grade tubular adenoma) composed of closely packed neoplastic glands with elongated nuclei. **D** Higher magnification of the NET component. Small clusters of neuroendocrine cells are seen immediately above muscularis mucosa. They are positive for serotonin (**D**, inset). (All sections from case 5)

The NET component was composed of uniform cells arranged in small nests or cords. NET components were also noted within the adenomatous glands at the basal side or were budded from the adenomatous glands. Neuroendocrine cells had nuclei with a salt-and-pepper pattern and indistinct nucleoli. The NET component did not exhibit mitosis or necrosis in any of the cases. Two intratumoral distribution patterns of the NET component were noted: (i) distribution from middle to deep lamina propria (basal–middle pattern; Fig. 1; *n* = 6) and (ii) distribution confined to deep lamina propria under the adenomatous component (deep lamina propria pattern; Fig. 2; *n* = 3). In two cases (cases 4 and 7), the horizontal spreading of the NET component was broader than that of the adenomatous component. The distribution pattern (basal–middle vs. deep lamina propria) was not associated with horizontal extension of the NET component or with tumor diameter.

None of the eight resected tumors exhibited vascular invasion. In all cases, the nonneoplastic background mucosa was gastric body mucosa exhibiting chronic atrophic gastritis with moderate/marked intestinal metaplasia and mucosal atrophy, a status compatible with the consequence of *H. pylori* gastritis. None of the cases had histopathological findings suggestive of autoimmune gastritis, such as enterochromaffin-like cell (ECL cell) hyperplasia. PPI-related changes, such as parietal cell protrusion or oxyntic gland dilataion, were also absent in the background mucosa.

### Immunohistochemical findings

Immunohistochemical analyses revealed that the NET component was diffusely positive for both chromogranin A and synaptophysin in all nine cases. In addition, these immunostainings highlighted linear or scattered neuroendocrine cells at the basal side of the adenoma component in eight of the nine cases. In six of the nine cases, the NET component was budding from the adenomatous component (Fig. 1E). In six of the seven assessed tumors, the NET component was diffusely positive for serotonin (Fig. 2D, inset) and showed an expression pattern of SSTR2A + /CDX2 + /VMAT2 − (Supplementary Fig. 1), suggesting they are serotonin-producing enterochromaffin-cell (EC-cell) phenotype [2] (Table 3, right). The NET component of a serotonin-negative case also showed a pattern of SSTR2A + /CDX2 + /VMAT2 − , which suggests EC-cell phenotype, though it lacked positivity for all the assessed hormones (serotonin, gastrin, somatostatin).

In all cases, the Ki-67 proliferation index was less than 2% in the NET component and was ranged between 2 and 30% in the adenomatous component. Regarding the cellular phenotype of the adenomatous component, six cases were classified as the intestinal type and two cases were classified as the mixed type (predominantly intestinal phenotype).

**Fig. 3:**
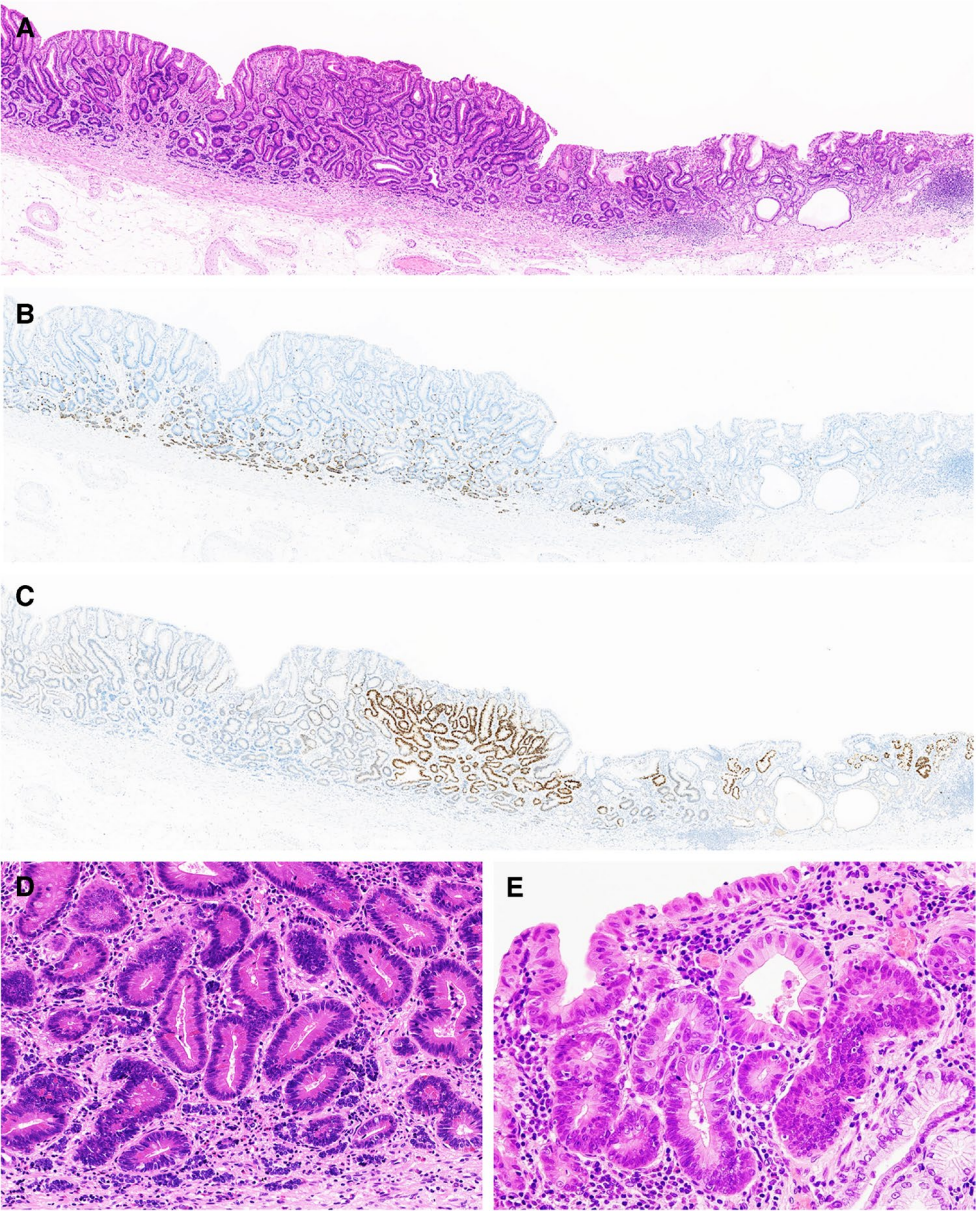
A case of gastric MANET with focal pattern of mutant p53 staining—case 8. **A**–**C** Loupe view of a section containing low-grade (left to center) and focal high-grade dysplasia component (right). Hematoxylin/eosin staining (**A**) shows that the area with high-grade dysplasia (right) is slightly depressed compared to the area with low-grade dysplasia. Chromogranin A staining (**B**) highlights the distribution of the NET component. **C** Immunohistochemistry for p53. Most adenoma glands (left) show wild-type p53 pattern, whereas focal high-grade dysplasia component (right) and adjacent periphery of low-grade component (center) show diffuse p53 positivity. The NET component shows wild-type p53 staining pattern. **D** Higher magnification of the MANET. Small vesicles composed of neuroendocrine cells intermingle with adenomatous glands in deep lamina propria. **E** Higher magnification of the focal high-grade dysplasia component composed of irregular glands with enlarged ovoid nuclei

Wild-type p53 staining pattern was observed in all but one case (case 8), in which mutant p53 pattern with diffuse and strong staining was noted in the high-grade adenoma component (Fig. 3). In none of the cases, nuclear β-catenin expression was observed in the adenomatous or the NET components.

### Review of the biopsy specimens

**Fig. 4:**
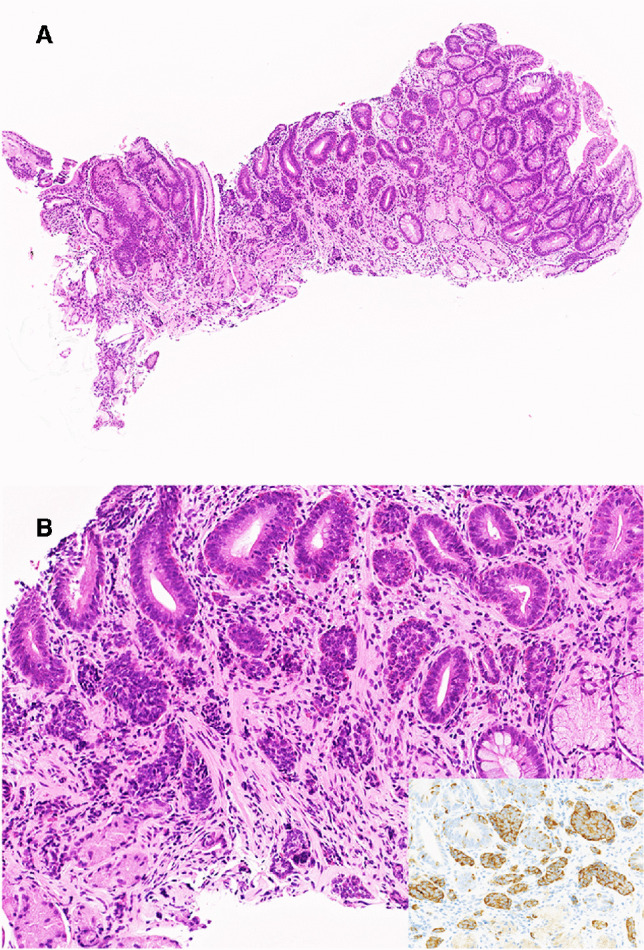
Prior gastric biopsy specimen of a patient later diagnosed with gastric MANET, basal–middle pattern—case 7. **A** Whole section of the biopsied specimen. The NET component, which forms nests smaller than adenomatous glands, is located in the center. **B** Higher magnification shows the NET component (inset, synaptophysin) together with adenoma glands

Preoperative biopsy specimens were available for review in six patients who underwent resection. Both the NET and adenomatous components were recognized in the biopsy specimens of two of the six cases (Fig. 4); in both cases, the NET component exhibited the basal–middle pattern of distribution.

### Follow-up data

Follow-up information was available in all nine patients, with follow-up duration ranging from 2 months to 8 years (mean, 30 months). One patient (case 9) died due to advanced colon cancer with urinary bladder invasion 2 months after the gastric biopsy. All of the remaining eight patients were alive at last-follow-up with no recurrence (Table 4).

**Table 4:** Summary of the literature review, including the patients with gastric MANET included in this study

References	Age (y)	Sex	Symptoms	Adenoma component			NET component			Metastasis	Recurrence (follow-up)
				Size (mm)	Structure	Grade	Size (mm)	Submucosal invasion	Grade		
Our case #1	61	M	None	35	Tubular	LG	17	No	G1	No	No (3 mo)
Our case #2	58	M	NA	32	Tubular	LG & focal HG	28	No	G1	No	No (2 mo)
Our case #3	80	F	NA	15	Tubular	LG	4	No	G1	No	No (2 mo)
Our case #4	64	M	None	44	Tubular	LG & focal HG	22	No	G1	No	No (5 y)
Our case #5	77	M	None	15	Tubular	LG	3	No	G1	No	No (8 y)
Our case #6	79	M	None	14	Tubular	LG	11	No	G1	No	No (3 y)
Our case #7	80	M	None	13	Tubular	LG	12	No	G1	No	No (4 y)
Our case #8	75	M	None	27	Tubular	LG & focal HG	14	No	G1	No	No (1 y)
Our case #9	75	M	None	8	Tubular	LG	NA	No	G1	No	DOC (2 mo)
Ito et al.^9^	54	M	None	20	Tubular	LG	4	No	NA	NA	No (19 y)
Harada et al.^8^	72	M	None	10	NA	NA	NA	No	NA	No	NA
De Marco et al.^7^	76	M	Dysphagia	10	Tubular	LG	2	No	G1	NA	No (10 y)
Coyne and O’Connor^6^	68	F	Abdominal discomfort	11	Tubular	LG	NA	Yes	G1	NA	NA
Lee et al.^10^	64	M	None	38	Tubular	NA	NA	No	NA	NA	No (2 y)
Lee et al.^10^	63	M	Abdominal discomfort	40	Tubular	NA	NA	No	NA	NA	No (2 y)
Lee et al.^10^	52	M	None	18	Tubular	NA	NA	No	NA	No	No (2 y)
Lee et al.^10^	65	M	Dyspepsia	41	Tubulovillous	HG	NA	Yes	NA	NA	No (12 y)
La Rosa et al.^3^	55	M	Dyspepsia	15	Tubular	HG	3	No	G1	No	No (27 y)

*NA*, not available; *LG*, low-grade; *HG*, high-grade; *DOC*, died of other cause

## Discussion

In the present study cohort, gastric MANETs were characterized by flat elevated lesions with a background of chronic atrophic gastritis. Histologically, the lesions were composed of intestinal-type tubular adenoma with low- or focal high-grade dysplasia, accompanied by grade 1 NET within the deep/middle lamina propria. By immunohistochemistry, p53 alterations were recognized only focally in the adenomatous component in one case. None of the patients developed recurrent disease after endoscopic resection during the follow-up period ranging from 2 to 94 months.

Gastric MANETs are extremely rare, with our literature search identifying only nine other reported cases (Table 4) [3, 6–10]. In line with our observations, the adenomatous component exhibited tubular structures in all previously reported cases save one case with tubulovillous adenoma. All 12 cases with available data were categorized as grade 1 NET (Table 4). The adenomatous component was usually low-grade, although a high-grade adenomatous component was also present in five cases. None of the patients with available follow-up data developed recurrent disease after resection (mean follow-up duration, 6 years; range, 2 months to 27 years). Based on these findings, gastric MANET is considered as an indolent neoplasm similar to colonic MANETs, distinct from MiNEN (adenocarcinoma-NEC or adenocarcinoma-NET). Therefore, endoscopic resection would be the primary therapeutic recommendation for gastric MANETs.

Given the indolent nature of gastric MANETs, pathologists should not misinterpret the NET component as nest-forming poorly differentiated non-neuroendocrine adenocarcinoma. When encountering nest-forming component in combination with adenomatous component, MANET should be included in the differential diagnosis.

The pathogenesis of gastric MANET is not fully understood. Some studies on intestinal MANETs [14, 15] have described the presence of a transition zone between the adenomatous and NET components, supporting the hypothesis that two components share the same origin. Lee et al. noted that neuroendocrine cells in gastric MANETs appeared to bud from the adenomatous gland [10]. In the present study, six of the nine cases harbored this budding-off phenomenon. We also noted clustered or linear endocrine cells along the basal side of the adenomatous glands in eight of the nine cases. Such neuroendocrine cells are likely to be a reservoir for the NET component observed in MANETs. As such, the present study findings further emphasize that the two components share the same origin.

Our patients did not have clinicopathological conditions associated with NET (i.e., autoimmune gastritis, ECL hyperplasia, MEN, or long-term PPI use). The endocrine component of MANETs is similar to type 3 NET in terms of the background condition and cellular phenotype. Based on our observations, however, the endocrine component of MANETs is likely to bud and emerge from adenomas and is therefore a distinct entity with a different origin from NETs. Thus, it is not surprising that the biological grade differs between MANETs and type 3 NETs, with the former being indolent and the latter aggressive [2].

In conclusion, our observations as well as the literature review support that gastric MANET is an indolent neoplasm similar to colonic counterpart. Pathologists should be aware of this entity to prevent overdiagnosis and overtreatment. Gastric MANETs may be treated conservatively, although endoscopic complete resection is recommended, because a subset of tumors includes high-grade adenomas.

## Supplementary Information

Below is the link to the electronic supplementary material.

Supplementary file1 (DOCX 550 KB)

## Data Availability

Data supporting the findings of this study are included in this published article and its supplementary information file.
