# Supplementary material for: Mixed adenoma-neuroendocrine tumor of the stomach: analysis of nine cases with literature review
Source: Virchows Arch. 2024 Jun 26;486(3):521–30. doi: 10.1007/s00428-024-03851-3 (PMC11950016; doi:10.1007/s00428-024-03851-3)
Supplement: Supplementary file 1 — Supplementary file1 (DOCX 550 KB) [file 428_2024_3851_MOESM1_ESM.docx]

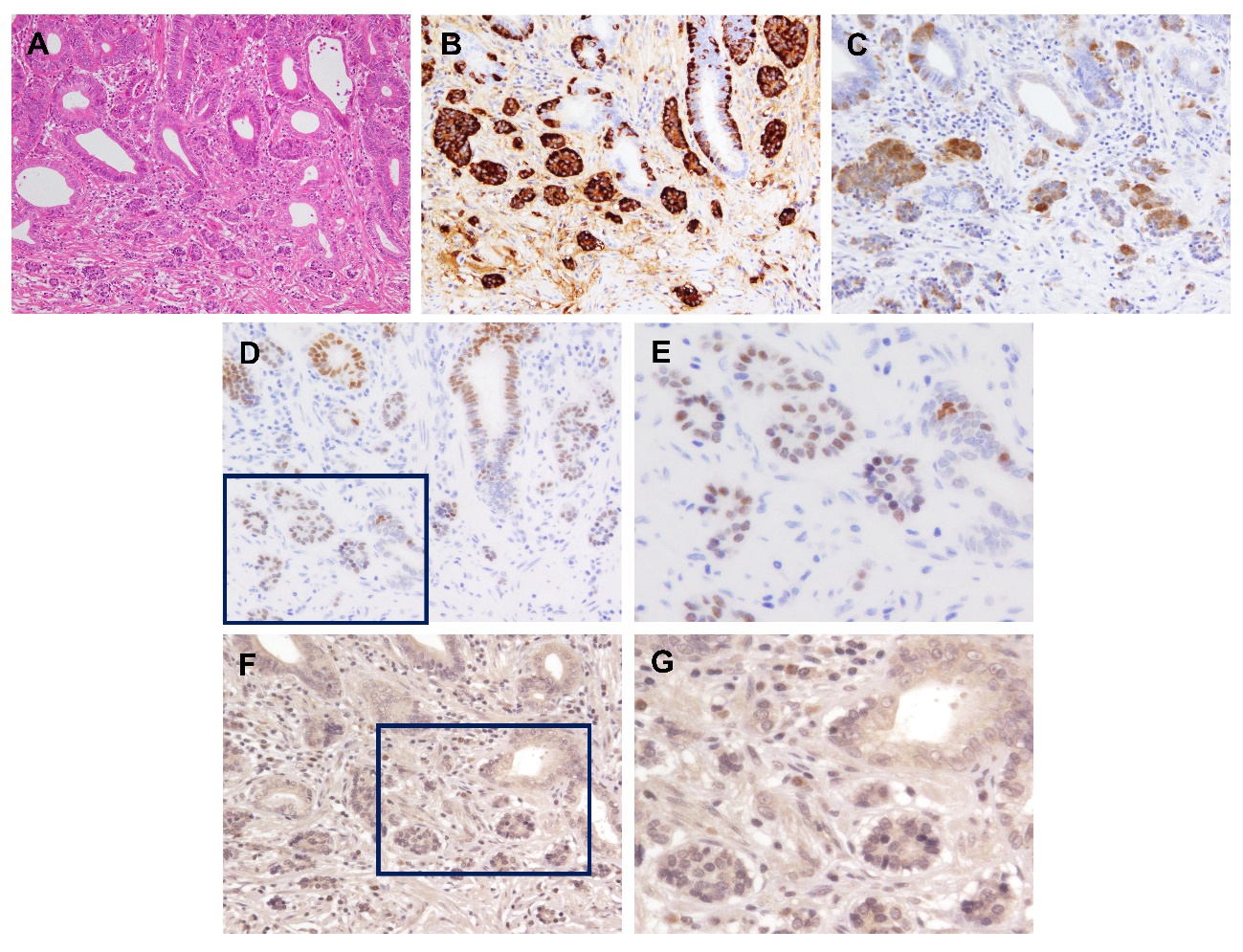


**Supplementary Figure 1.** Detailed immunohistochemical profile of a representative case (#7).

This MANET (A, HE staining) harbors NET component positive for chromogranin (B) and serotonin (C). NET component showed positive nuclear CDX2 expression (D and E) but was negative for VMAT2 (F and G). (E) and (G) are high-power views of the frame in (D) and (F), respectively. These findings suggest that NET cells are of EC-cell phenotype. VMAT2 immunohistochemistry was conducted using normal adrenal medulla as a control.
